# Severe Hypercalcaemia – Chronic Tophaceous Gout as the Responsible Cause?

**DOI:** 10.17925/EE.2015.11.02.102

**Published:** 2015-08-19

**Authors:** René Rodríguez-Gutiérrez, Maria Azucena Zapata-Rivera, Karla Victoria Rodriguez-Velver, Fernando J Lavalle-Gonzalez, José Gerardo Gonzalez-Gonzalez, Jesus Zacarias Villarreal-Perez

**Affiliations:** 1. Endocrinology Division, Department of Internal Medicine, University Hospital ‘Dr José E González’, Unlversldad Autonoma de Nuevo León, Monterrey, México; 2. Knowledge and Evaluation Research Unit, Division of Endocrinology, Diabetes, Metabolism and Nutrition, Department of Medicine, Mayo Clinic, Rochester, Minnesota, US

**Keywords:** Tophaceous gout, severe hypercalcaemia, calcitriol, thopi

## Abstract

The association of chronic tophaceous gout with severe hypercalcaemia is exceptional. In this case, a 42-year old man with a long-standing history of gout arrived at the emergency room with altered mental status. Laboratory work up revealed a uric acid of 14.0 mg/dl, corrected calcium of 14.5 mg/dl, phosphorous of 6.3 mg/dl, parathyroid hormone (PTH) was suppressed (<3.0 pg/ml), 25-dihydroxyvitamin D 25.2 ng/ml, parathyroid hormone related-protein (PTHrP) was 45.0 pg/ml and calcitriol 19.6 pg/ml. Biopsy histopathology result showed deposits of monosodium urate crystals surrounded by granulomatous inflammation. The association of chronic tophaceous gout with severe hypercalcaemia is extremely rare and has been usually described to be secondary to 1-25 dihydroxyvitamin D (calcitriol) secretion. In this case, calcitriol levels were normal and this possibility was excluded. On the other hand, PTHrP had never been, until now, described as the responsible cause of hypercalcaemia in gout. In our case, baseline PTHrP and calcium values were elevated and after medical treatment both returned to normal values. PTHrP usually causes hypophosphataemia and in this case the abnormal renal function could have diminished this last effect.

## Case Presentation

Sometimes the worst has to happen before seeking medical attention. This was the case of a 42-year-old man who arrived to the emergency room with altered mental status characterised by lethargy and confusion. His sister referred a long-standing history of gout that was diagnosed 8 years earlier and that was treated irregularly with non-steroidal anti-inflammatory drugs (NSAIDs) and colchicine and reported a negative family history of gout. Two years earlier, multiple tophi had appeared on the ears, elbows, hands and foot and 6 months previously he was confined to bed due to intense generalised pain. Despite his family insistence, the patient constantly refused to receive medical attention. A week earlier he referred initiating with polyuria, polypsia and constipation and nausea and vomiting developed 48 hours before admission. Progressive altered mental status finally made his family pursue formal medical attention.

## Assessment

**Figure 1:**
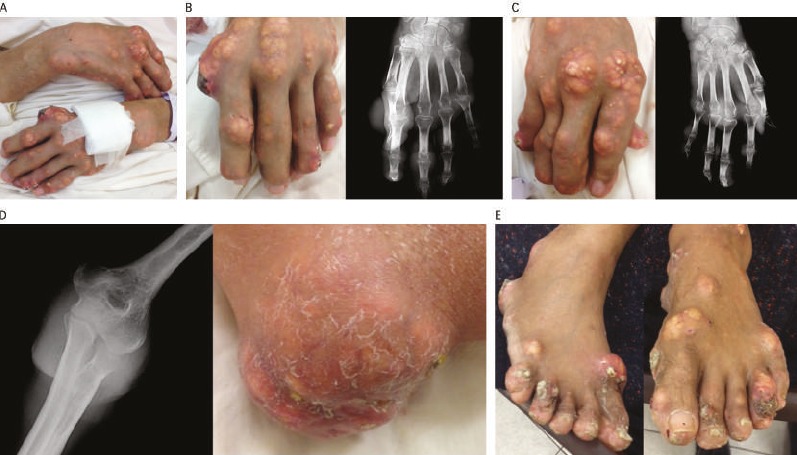
Physical Examination Findings and Description Non-tender tophi from 1 to 6 cm were obvious at inspection. A–C. Wrists, metacarpophalangeal, proximal and distal interphaiangeal articulations. D. Elbows. E. Ankles and first metatarsophalangeal joint. Radiographs of upper and lower extremities revealed bone erosions with the characteristic ‘overhanging’ edges.

Physical examination revealed altered mental status characterised by lethargy and a Glasgow coma scale of 14 without any signs of focalisation. Blood pressure was 130/85 mmHg, respiratory rate 13 per minute, heart rate 95 beats per minute, temperature 36.3°C and room-air oxygen saturation was 98 %. Characteristic, multiple, non-tender tophi from 1 to 6 cm were obvious at inspection in the wrists, metacarpophalangeal, proximal and distal interphalangeal articulations, elbows, knees, ankles and the first metatarsophalangeal joint (see *Figure 1*). A non-infected ulcer was seen in the second metacarpophalangeal joint. There were no signs of septic arthritis and the rest of the examination was unremarkable.

Laboratory work up revealed a uric acid of 14.0 mg/dl, corrected calcium of 14.5 mg/dl, phosphorous of 6.3 mg/dl, creatinine of 5.4 mg/dl, blood nitrogen urea of 56, a Modification of Diet in Renal Disease (MDRD) glomerular filtration rate (GFR) of 16 ml/minute and urinary calcium of 350 mg/24 hours. PTH was suppressed (<3.0 pg/ml), 25-dihydroxyvitamin D was normal, parathyroid hormone related-protein (PTHrP) was 45.0 pg/ ml and calcitriol 19.6 pg/ml. Red blood cell count was normal and a peripheral smear showed no dysmorphic red cells. Twenty-four hour protein excretion was 110 mg/day (see *Table 1*). An electrocardiogram showed the characteristic shortened QT interval with no other rhythm abnormalities. Radiographs of upper and lower extremities revealed bone erosions with the characteristic ‘overhanging’ edges (see *Figure 1*). Biopsy histopathology result showed deposits of monosodium urate (MSU) crystals surrounded by granulomatous inflammation.

## Diagnosis

The most common cause of severe hypercalcaemia in a hospitalised setting is malignancy. The responsible mechanisms are known to be secondary to local osteolysis, PTHrP mediated, 1,25-dihydroxyvitamin D (1,25(OH)_2_D_3_, calcitriol) secretion and ectopic parathyroid hormone (PTH).^1^ PTHrP as seen in our patient, is the most common cause and has usually been described in non-metastatic solid tumours (bladder, breast and squamous cell tumors) and non-Hodgkin lymphomas. In this sense, a bone scan and a positron emission tomography-computed tomography (PET-CT) were negative for metastasis and malignancy. Nevertheless, usually associated with mild/moderate levels of hypercalcaemia, hyperparathyroidism, overall, is known to be the most common cause of hypercalcaemia and was also ruled out.^2^ As causes of secondary hyperucricaemia, drugs and/or diet supplements were excluded, as well as haemolytic disorders, obesity, psoriasis and myeloproliferative and/or lymphoproliferative disorders. There was no family history consistent with an inherited defect that could lead to purine overproduction.

The association of chronic tophaceous gout with severe hypercalcaemia is extremely rare and has been usually described to be secondary to 1-25 dihydroxyvitamin D (calcitriol) secretion. The proposed mechanism has been an enhanced 1α-hydroxylation of vitamin D in a proliferative chronic synovitis with granulomatous inflammation and giant cells (macrophages), with a consequent increase in active calcitriol that results in hypercalcaemia and/or hypercalciuria.^3,4^ In this case calcitriol levels were normal and this possibility was excluded. In addition to this, after treatment with prednisone the levels of calcitriol were no different. On the other hand, PTHrP had never been, until now, described as the responsible cause of hypercalcaemia in gout. In our case, baseline PTHrP and calcium values were elevated and after medical treatment both returned to normal values. PTHrP usually causes hypophosphataemia and in this case the abnormal renal function could have diminished this last effect. Immobilisation is another well-known cause of mild calcium elevations.^5^ It is likely that in this case this was an exacerbating rather than the primary factor. Local osteolysis was considered but even in advanced rheumatoid arthritis and other severe causes of bone erosions, hypercalcaemia has never been reported.

**Table 1:** Laboratory Measures

Value	Basal	Post-treatment	Range
Haemoglobin (g/dl)	15.8	13.5	(12–16)
Haemoglobin (mmol/l)	9.8	8.4	(7.4–9.9)
Uric acid (mg/dl)	14.0	8.2	(0–7)
Uric acid (umol/l)	832.7	487.7	(0–416)
Glucose (mg/dl)	84	82	(70–100)
Glucose (mmol/l)	4.7	4.6	(3.9–5.6)
Creatinine (mg/dl)	5.4	0.8	(0.6–1.2)
Creatinine (umol/l)	477.3	70.7	(53–106)
Urea nitrogen (mg/dl)	56	17	(8–23)
MDRD GFR (ml/minute)	12.0	96.1	(≥60)
Albumin (g/dl)	3.1	3.7	(3.5–5.0)
Albumin (g/l)	3.1	37	(35–50)
Calcium (mg/dl)	14.5	9.6	(8.2–10.2)
Calcium (mmol/l)	3.6	2.4	(2.0–2.55)
Phosphate (mg/dl)	6.3	3.7	(2.3–4.7)
Phosphate (mmol/l)	2.0	1.2	(0.7–1.5)
Magnesium (mg/dl)	1.8	2.3	(1.5–2.3)
Magnesium (mmol/l)	0.74	0.95	(0.62–0.95)
Potassium (meq/l)	3.9	4.3	(3.5–5.0)
Urinary calcium (mg/day)	350	150	(100–250)
Urinary protein (mg/day)	112	98	(≤150)
Alkaline phosphatase (UI/l)	175	134	(50–120)
PTH (pg/ml)	<3.0	15.7	(1.5–37)
PTH (pmol/l)	<0.31	1.66	(0.15–3.92)
PTHrP (pg/ml)	45.0	9.7	(14–27)
PTHrP (ng/l)	45.0	9.7	(14–27)
25(OH)D_3_ (ng/ml)	25.2	27.5	(>20)
25(OH)D_3_ (nmol/l)	63.9	68.6	(>49.9)
1,25(OH)_2_D_3_ (pg/ml)	19.6	23.7	(18–38)
1,25(OH)_2_D_3_ (pg/ml)	47.0	56.9	(43.2–91.2)

*1,25(OH)_2_D_3_ = 1,25-dihydroxyvitamin D; 25(OH)D_3_ = 25,Hydroxyvitamin D; LDH = lactate dehydrogenase; MDRD GFR = Modification of Diet in Renal Disease glomerular filtration rate; PTH = parathyroid hormone; PTHrP = parathyroid hormone related protein*.

## Management

Now rarely seen, chronic tophaceous gout represents the last stage of the natural history of gout. It is characterised by collections of solid urate in connective tissues such as cartilage, bursae, soft tissues, tendons, ligaments and enthesis. It is composed of deposits of MSU crystals that are usually surrounded by granulomatous inflammation.^1^ Although clinical signs of acute inflammation sometimes occur in tophi, this is unusual; swelling accompanying a tophus is most often due to the mass of crystals itself.^2^

Treatment was initiated with calcitonin, hydration with intravenous saline solution at 250 ml/hour and prednisone with a consequent improvement in renal function and normocalcaemia restoration.^6^ PTH, 25-dihydroxyvitamin D, PTHrP and calcitriol returned to normal values and after 3 weeks, the patient was discharged with prednisone, NSAIDs and colchicine. At 6 months follow-up the tophi had improved, calcium levels were within the normal range and no pain was reported. In this case the overproduction of PTHrP and the immobilisation were the responsible mechanisms of the hypercalcaemia that was restored after prompt and optimal treatment.
